# Spontaneous Bilateral Meningoencephalocoeles of the Temporal Bones

**DOI:** 10.1155/2013/969762

**Published:** 2013-10-29

**Authors:** Oliver Rose, Michel Neeff, Christopher Low

**Affiliations:** ^1^Auckland City Hospital, Auckland 1042, New Zealand; ^2^Rotorua Hospital, Rotorua, New Zealand

## Abstract

Spontaneous tegmen tympani defects are rare with even rarer bilateral cases. The symptoms are nonspecific; hence, a high index of suspicion is required to prevent serious intracranial complications. We present a case of spontaneous bilateral tegmen tympani defects with associated meningoencephalocoeles in a 54-year-old male who presented with the signs and symptoms of severe meningitis. After careful workup which included a lumbar puncture, CT and MRI scans, both defects were repaired using a middle fossa approach. The patient made an uneventful recovery with complete cessation of otorrhoea and improvement in his hearing.

## 1. Introduction

CSF leaks from skull base defects are classified as either spontaneous with no obvious cause or secondary due to trauma, surgery, tumours, infection, and inflammation [1, 2]. Patients may be asymptomatic or present with intracranial complications such as meningitis. Meningoencephalocoeles of the temporal bone can herniate through the tegmen tympani. If there is a defect into the CSF space, patients may present with CSF otorrhoea in the presence of a perforated tympanic membrane or CSF rhinorrhoea through the Eustachian tube [3]. A conductive hearing loss may be due to a CSF middle ear effusion. Spontaneous unilateral tegmen tympani defects are uncommon; bilateral defects are rarely encountered [4]. There are distinct patient groups who are at risk of meningitis from acute otitis media: children with congenital inner ear abnormalities (e.g., Mondini dysplasia), dehiscent stapes footplate, tympanomeningeal fistula, patent fallopian canal, and middle-aged adults with no identifiable congenital or acquired causes [1, 2, 5]. Aetiological theories of idiopathic intracranial hypertension and arachnoid granulations in bony erosions have been suggested [6, 7]. We present a case of bilateral meningoencephalocoeles of the temporal bones with CSF leak and review the literature of this entity. 

## 2. Case Report

A 54-year-old man presented to the emergency department with agitation and seizures following a generalized febrile illness and a progressively worsening headache over 24 hours. He was treated with ceftriaxone IV, metronidazole, and acyclovir. A contrast CT scan of the brain showed no focal central lesion but features in keeping with bilateral chronic middle ear and mastoid disease. A lumbar puncture showed no evidence of raised intracranial pressure (ICP). CSF microscopy supported the diagnosis of meningitis, and on culture streptococcus sensitive to ceftriaxone and amoxicillin was grown. He responded to a three-week course of appropriate systemic antibiotics and made a complete recovery. On reviewing his history, he had been able to produce clear rhinorrhoea on leaning forward for, years and this tested positive for beta-2 transferrin. He had a left conductive hearing loss due to a middle ear effusion for several years. He had a grommet inserted in 2008, which was later removed due to persistent clear otorrhoea. CSF analysis at the time was inconclusive, and a CT scan of the temporal bones reportedly showed no abnormality. In 2000, he had a generalized seizure for which no cause could be found, and he has been on phenytoin ever since. He has type 2 diabetes mellitus for which he is on insulin. There was no past history of head trauma, CNS infections, neurologic, and otologic surgery. A High resolution CT and MRI scans of the petrous temporal bones were performed confirming bilateral tegmen tympani dehiscence, bilateral meningoencephalocoeles and bilateral middle ear and mastoid effusions. 

A middle fossa approach was used to repair both defects starting with the left. Six months later, the contralateral side was repaired. A 20 × 10 mm defect in the tegmen with substantial herniation of brain tissue was noted on the right, and an 8 × 2 mm defect with encephalocoele was present on the left. A strip of cortical bone harvested from the bone flap was placed over the defects which were then covered with a fascia lata graft. Tisseel was used to secure the graft in place. The recovery period was uneventful. CSF leakage ceased after surgery, and his hearing improved bilaterally.

## 3. Discussion

A defect in the tegmen tympani can result in CSF otorrhoea in the presence of a tympanic membrane perforation or a ventilation tube, conductive hearing loss, and aural fullness with an intact tympanic membrane. The findings of clear, watery, and pulsatile middle ear fluid at the time of myringotomy for conductive hearing loss secondary to a middle ear effusion are well documented [4, 8]. If the tympanic membrane is intact, the patient may report clear rhinorrhoea or postnasal drip due to passage of CSF from the middle ear to the nasopharynx via the Eustachian tube. Otorrhoea occurs when there is a breach in the region of the temporal bone, whereas rhinorrhoea can also be associated with an anterior skull base defect [3, 9]. 

Less commonly, a middle ear mass due to the prolapse of a cephalocoele may be the initial presentation [9, 10]. A skull base cephalocoele occurs when intracranial content herniates through a skullbase defect. This can involve the meninges alone (meningocoele) or include herniation of brain (encephalocoele). Similar to CSF leaks, cephalocoeles are also classified as congenital, spontaneous, or secondary [2]. 

There is still much controversy as to what causes spontaneous tegmen tympani defects with various theories being postulated. Cases presenting in the paediatric population associated with anomaly of the inner ear and congenital hearing loss are thought to be caused by abnormal embryologic development, which results in gaps within the skull base [2, 7, 11]. It is now increasingly recognised that there is a group of patients with skull base defects that present later in life with no associated congenital anomalies. The most widely accepted theory suggests the formation of aberrant arachnoid granulations which promotes bony erosion especially adjacent to pneumatised areas of the skull base. This is supported by the fact that areas lateral to the cribriform plate and along the floor of the middle cranial fossa are common sites for aberrant arachnoid granulations [1, 12]. There may also be an association between benign intracranial hypertension and spontaneous CSF leaks. This has been mainly observed in an obese middle-aged woman [6, 7].

Recognition of a tegmen tympani defect is important because of the potential of developing meningitis and other intracranial complications. The index of suspicion should be higher in cases of recurrent meningitis [11]. Any obvious associated clear otorrhoea should be tested for beta-2 transferrin which is highly specific for human CSF and perilymph [1, 9]. However, a negative beta-2 transferrin test does not rule out a CSF leak. Appropriate imaging may help make the diagnoses in these patients [13]. 

**Figure 1:**
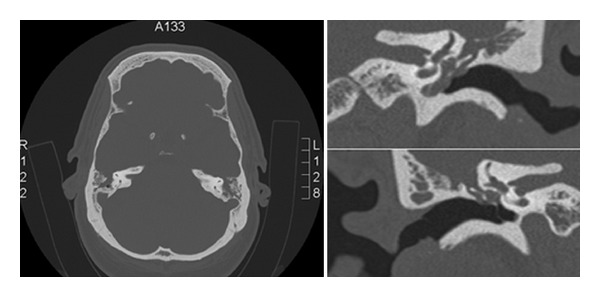
Axial and coronal CT images showing the tegmen defects.

As seen in this case, a CT of the brain done prior to performing a lumbar puncture may fail to identify skull base defects. High resolution CT images of the temporal bones with 1 mm slices scanned in both axial and coronal planes are recommended (Figure 1). MRI is useful in defining any soft tissue structures such as tumours, inflammatory tissue, cholesteatoma, and cephalocoeles and can also show radiological signs of idiopathic intracranial hypertension which may be associated with spontaneous CSF leaks [2]. 

In our case, even though there were clinical features of a CSF leak in 2008, CT scan of the temporal bones failed to show the defect. Studies have shown that high resolution CT can identify the associated skull base defect in most but not all cases of CSF leak [14]. We can assume that the defects were small enough initially to be missed on the CT scan at the time. It is also important to assess both sides of scans as some defects may be asymptomatic on presentation and overlooked as a result.

For hearing preservation and with the relative anterior location of the tegmen, a middle cranial fossa approach was used to repair both defects. This approach seems to be the consensus in patients with tegmen tympani defects and serviceable hearing [5]. The optimal approach for the management of tegmen mastoideum and posterior fossa defects is still unclear with transmastoid, middle cranial fossa, and combined approaches being advocated by various groups [9, 15, 16]. 

## 4. Conclusion

**Figure 2:**
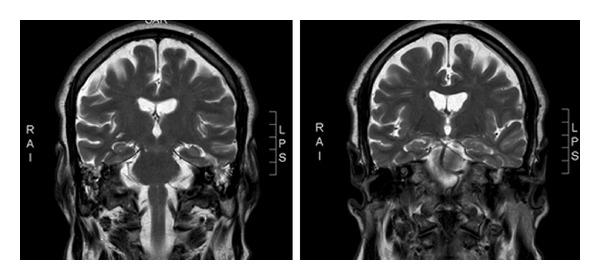
MRI showing areas highly suspicious of herniation. This was confirmed intraoperatively.

A spontaneous tegmen tympani defect is a rare but an important diagnosis given the potential for catastrophic intracranial sepsis. Diagnosis is based on clinical signs and symptoms such as clear rhinorrhoea/otorrhoea and conductive hearing loss. However, certain patients can present in extremis with meningitis or other intracranial complications. High resolution CT with 1 mm slices is essential for locating the defect. MRI scan is helpful to detect herniation of a meningoencephalocoele (Figure 2) and associated intracranial pathology. Beta-2 transferrin testing is extremely useful due to its high sensitivity and specificity for CSF and perilymph. If the index of suspicion remains high, despite normal findings of these investigations, a repeat may be warranted.
